# Pronostic rénal de la néphropathie des vascularites à IgA de l’adulte: étude monocentrique, à propos de 25 cas

**DOI:** 10.11604/pamj.2018.31.9.10594

**Published:** 2018-09-04

**Authors:** Amel Harzallah, Hayet Kaaroud, Narjess Laadhari, Rim Goucha, Ezzeddine Abderrahim, Sami Turki, Fethi Ben Hmida, Samia Barbouch, Taieb Ben Abdallah

**Affiliations:** 1 Service de Médecine Interne A, Hopital Charles Nicolle, Tunis, Tunisie; 2 Faculté de Médecine de Tunis, Université de Tunis El Manar, Tunis, Tunisie; 3 Laboratoire de Recherche de Pathologie Rénale, Hôpital Charles Nicolle, Tunis, Tunisie

**Keywords:** Purpura, néphropathie, pronostic, Purpura, nephropathy, prognosis

## Abstract

La néphropathie de la vascularite à IgA conditionne le pronostic de cette affection chez l’adulte. Le but de notre étude était d’étudier les caractéristiques cliniques de cette atteinte rénale chez l’adulte et d’identifier les facteurs de pronostic rénal. Il s’agit d’une étude monocentrique rétrospective portant sur les patients ayant une vascularite à IgA (purpura rhumatoïde) (critères de l’EULAR) avec une atteinte rénale prouvée histologiquement et classée selon la classification de Pillebout. Nous avons analysé la survie rénale et identifier les facteurs de pronostic rénal. Vingt cinq patients ont été inclus (genre ratio M/F = 2,57) d’âge moyen au diagnostic du purpura rhumatoïde de 35,76 ans. Un purpura était présent dans 100% des cas avec une atteinte articulaire dans 28%. Une insuffisance rénale était présente dans 44% des cas. La classification histologique la plus fréquente était la classe II. Une rémission clinique a été observée dans 44% des cas et une évolution vers le stade terminal de l’insuffisance rénale chronique dans 36% des cas. La survie rénale à 195 mois était de 57%. Les facteurs pronostiques identifiés étaient l’atteinte digestive (p = 0,022), l’insuffisance rénale initiale (p = 0,0004), la classification glomérulaire (p = 0001) et la sévérité des lésions histologiques, le traitement par bloqueurs du système rénine angiotensine (p = 0,01) et les échanges plasmatiques (p = 0,03). Notre étude montre que l’atteinte rénale au cours des vascularites à IgA peut être relativement sévère avec un mauvais pronostic rénal. L’identification des facteurs pronostiques cliniques et histologiques pourrait guider l’élaboration d’études thérapeutiques prospectives.

## Abstract (en)

IgA vasculitis nephritis affects the prognosis of this disease in adult patients. This study aimed to examine the clinical characteristics of this renal involvement in adults and to identify factors influencing renal prognosis. We conducted a retrospective monocentric study of patients with histologically confirmed IgA vasculitis nephritis (rheumatoid purpura) (EULAR classification criteria) with renal involvement classified according to Pillebout classification. We analyzed renal survival and identified the factors influencing renal prognosis. Twenty-five patients were included (sex ratio M/F = 2.57), their average age at diagnosis of rheumatoid purpura was 35,76 years. Purpura was diagnosed in 100% of cases, with articular involvement in 28% of cases. Renal failure was identified in 44% of cases. The most common histological classification was IgA vasculitis nephritis (class II). Clinical remission was observed in 44% of cases and an evolution toward chronic renal failure (end-stage renal disease) in 36% of cases. Renal survival at 195 months was 57%. The identified prognostic factors were digestive involvement (p = 0.022), early renal failure (p = 0.0004), glomerular classification (P=0,001) and the severity of the histological lesions, renin-angiotensin system blocker treatment (p = 0.01) and plasma exchanges (p = 0.03). Our study shows that renal involvement during IgA vasculites can be relatively severe with poor renal prognosis. The identification of clinical and histological prognostic factors may be useful as guidance for the development of prospective therapeutic studies.

## Introduction

La vascularite à IgA (anciennement purpura rhumatoïde) est une vascularite systémique des petits vaisseaux en rapport avec des dépôts de complexes immuns contenant des immunoglobulines A [1]. Cette nouvelle nomenclature a été retenue lors de la conférence de consensus révisé de Chapel Hill en 2012 [2]. Elle est caractérisée par l’association de signes cutanés, articulaires et gastro-intestinaux pouvant survenir par poussées successives [1]. L’atteinte rénale peut s’associer à ces manifestations et conditionne le pronostic à long terme [3, 4]. Cette vascularite est plus fréquente chez l’enfant par rapport à l’adulte chez lequel elle se distingue par un polymorphisme clinique et une sévérité particulière [5, 6]. L’incidence de l’atteinte rénale chez l’adulte varie de 45 à 80% selon les séries avec un risque de progression vers l’insuffisance rénale plus fréquent chez l’adulte par rapport à l’enfant [3, 7-10]. Le but de cette étude était de décrire les caractéristiques, cliniques, biologiques et histologiques de la néphropathie de la vascularite à IgA et d’identifier les facteurs de pronostic rénal avec étude de la survie rénale.

## Méthodes

Il s’agit d’une étude descriptive, rétrospective, analytique et monocentrique concernant les patients pris en charge dans notre unité pour une vascularite à IgA avec une atteinte rénale pour laquelle ils ont bénéficié d’une ponction biopsie rénale durant la période allant de à 1990 à 2015. Les patients inclus devaient être âgés de plus de 16 ans, remplir les critères diagnostiques de l’EULAR [11] et présenter des anomalies rénales indiquant la pratique d’une ponction biopsie rénale: hématurie et/ou protéinurie et/ou insuffisance rénale. La présence de dépôts mésangiaux d’IgA en immunofluorescence à l’histologie rénale était exigée dans l’inclusion des malades. On n’a pas inclu les patients présentant une néphropathie à IgA sans signes systémiques (maladie de Berger), ceux ayant d’autres affections associant une néphropathie à un purpura vasculaire (lupus érythémateux systémique, cryoglobulinémie) ainsi que ceux ayant une thrombopénie (plaquettes < 100000). Les patients ayant une vascularite à IgA secondaire à une hépatopathie ou une maladie digestive ou articulaire inflammatoire chronique ont été également exclus de l’étude.

Les données ont été recueillies à partir des dossiers médicaux. Nous avons précisé l’âge au diagnostic, les manifestations extra rénales et les données biologiques. Le dosage des IgA sériques a été noté quand il était disponible. La fonction rénale a été évaluée par la formule MDRD. L’insuffisance rénale chronique terminale (IRCT) a été définie par une clairance rénale de la créatinine inférieure à 15 ml/min avec le recours à l’épuration extrarénale en l’absence d’un facteur de décompensation aigue. L’étude histologique au microscope optique ainsi que l’immunofluorescence ont été réalisés au laboratoire d’anatomo-pathologie de notre unité. Les lésions histologiques ont été classées selon la classification de Pillebout [**3**]. Un minimum de 10 glomérules était exigé. On a évalué la prolifération endocapillaire et son étendue ainsi que la prolifération extracapillaire et au niveau de l’interstitium la présence d’une fibrose ou inflammation interstitielles. Les proportions des glomérules comprenant des croissants, de la nécrose fibrinoïde et une sclérose globale ont été précisées.

### Analyse statistique

Les données ont été saisies au moyen du logiciel Excel 2007 et analysées au moyen du logiciel SPSS version 19.0. Nous avons calculé des fréquences absolues et des fréquences relatives (pourcentages) pour les variables qualitatives. Nous avons calculé des moyennes, des médianes et des écarts-types et déterminé les valeurs extrêmes pour les variables quantitatives. La recherche des facteurs pronostiques de survie a été effectuée en analyse univariée (facteur par facteur) en comparant les courbes de survie par le test du Log rank. Une différence est déclarée significative à chaque fois que la valeur de p est inférieure à 0,05. Les données de survie ont été étudiées en établissant une courbe de survie selon la méthode de Kaplan Meier.

## Résultats

**Figure 1:**
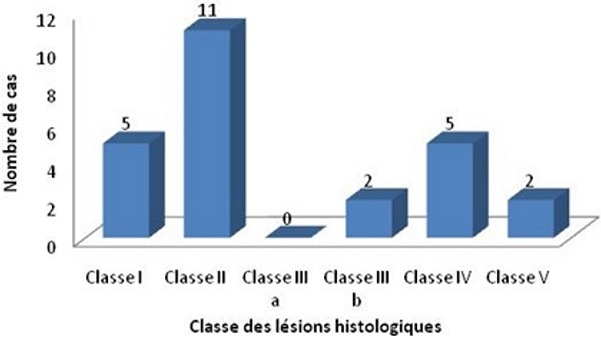
Répartition des lésions histologiques des patients

Vingt cinq patients ayant une vascularite à IgA (V IgA) avec une atteinte rénale ont été inclus. Il s’agissait de 1^8^ hommes et t femmes avec un genre ratio M/F égal à 2,57, d’âge moyen au diagnostic de la vascularite de 35,76 ans [16-69 ans]. La néphropathie était concomitante au diagnostic de la V IgA dans 19 cas et elle est survenue après un délai moyen de 20,16 mois [2-108 mois] dans 6 cas (24%). Un facteur infectieux déclenchant a été retrouvé dans 6 cas (24%). Un tabagisme a été noté dans 9 cas (36%). Les caractéristiques cliniques et biologiques des patients sont résumées dans le Tableau 1 et Tableau 2. Une HTA était présente dans 9 cas (36%). Le taux de BMI moyen était de 22,64 kg/m^2^ [16-33,77]. La fonction rénale était normale dans 14 cas (56%). Une clairance de la créatinine inférieure à 60 ml/mn a été notée dans 11 cas (44%). Une protéinurie était notée dans 21 cas (84%) avec un syndrome néphrotique dans 7 cas (28%). Un syndrome néphrétique aigu était présent dans 3 cas (12%). Le dosage des Ig A sériques réalisé dans 9 cas (36%) a été retrouvé à un taux élevé dans 2 cas (8%). L’analyse histologique de la biopsie cutanée a été réalisée dans 14 cas (56%) concluant à une vascularite leuco-cytoclasique dans 7 cas (28%) avec des dépôts d’Ig A dans 4 cas (16%). La lésion la plus fréquente à l’histologie rénale était la glomérulonéphrite segmentaire et focale (classe II) retrouvée dans 11 cas (44%) (Figure 1). Les différentes lésions histologiques retrouvées ont été synthétisées dans le Tableau 3.

**Tableau 1:** Caractéristiques cliniques des patients

Paramètres	Nombre de cas	Pourcentage (%)
Fièvre	8	32
Purpura	25	100
Arthralgies	23	92
Arthrites	7	28
Douleurs abdominales	20	80
Vomissements	5	20
Hémorragie digestive	4	16
Céphalées	5	20
Convulsions	2	8
Œdèmes	19	76
Hématurie micro	25	100
Hématurie macro	8	32

Micro: microscopique, macro: macroscopique

**Tableau 2:** Paramètres biologiques des patients

Paramètres	Valeur moyenne	Extrêmes
Urémie (mmol/l)	16,55	2-52
Créatinine sanguine (µmol/l)	309,80	43-1461
Cl créat (ml/mn)	75,8	3,9-223
Uricémie (µmol/l)	386	152-720
Protidémie (g/l)	63,04	40-77
Albuminémie (g/l)	29,05	17-47,3
Calcémie (mmol/l)	2,17	1,51-2,48
VS (mm à la 1^ère^ heure)	71,78	11-150
CRP (mg/l)	29,89	1-111
LDH (UI/l)	500,4	157-1054
Protéinurie (g/24h)	3,26	0-8,8

Cl créat: clairance de la créatinine, VS: vitesse de sédimentation, CRP: protéine C réactive, LDH: lacticodéshydrogénase

**Tableau 3:** Lésions histologiques retrouvées chez nos patients

Paramètres	Nombre	Pourcentage (%)
Prolifération endocapillaire	9	36
Inflammation interstitielle	6	24
Intégrité de la capsule de Bowman	20	80
Croissants	10	40
Fibrose interstitielle	13	52
Atrophie tubulaire	4	16
Nécrose glomérulaire>10%	5	20
Sclérose glomérulaire>10%	5	20
Fibrose interstitielle >10%	8	32
Cylindres hématiques	7	28

Le traitement a comporté une corticothérapie orale dans 17 cas (68%) à une dose variant entre 0,5 et 1,5 mg/kg/j associée à un traitement immunosuppresseur à base de cyclophosphamide dans 1 cas et de chloraminophène dans un autre cas. Un traitement par colchicine a été associé dans un cas. Des échanges plasmatiques ont été réalisés dans deux cas. Un traitement par bloqueur du système rénine angiotensine aldostérone (BSRAA) a été reçu dans 8 cas (32%). Une PBR itérative a été réalisée dans deux cas devant l’aggravation de la fonction rénale concluant à un passage d’une classe II à une classe IV dans les deux cas. Apres un suivi moyen de 79,62 mois [3-228], l’évolution était marquée par une rémission clinique dans 11 cas (44%), une dégradation de la fonction rénale avec recours à l’épuration extra rénale définitive dans 9 cas (36%) après un délai moyen de 6,55 mois [0-18]. Deux patients ont bénéficié d’une transplantation rénale après un délai de 6 et 27 mois respectivement. Le premier patient a présenté une poussée de la maladie sous corticoïdes et ciclosporine après 13 mois de la greffe avec une atteinte rénale. L’histologie rénale a conclu à une classe II de la V IgA. Il est décédé au décours d’un sepsis sévère avec des lésions d’angéite. Sa fonction rénale était altérée à son décès. Le deuxième patient est décédé également après un délai de 228 mois en raison d’une ischémie myocardique avec arrêt cardio-respiratoire. Il avait une insuffisance rénale aigue en rapport avec une thrombose de l’artère du greffon rénale sous corticoïdes et azathioprine.

Quatre patients ont été perdus de vue. La survie rénale à 195 mois était de 57%. Après analyse univariée, les facteurs de pronostic rénal identifiés étaient l’atteinte digestive et notamment les vomissements (p = 0,022) ainsi que l’insuffisance rénale initiale (p = 0,0004). La classification glomérulaire a été également identifiée comme un facteur pronostique (p = 0,0001) ainsi que la sévérité des lésions histologiques: la prolifération endocapillaire (p = 0,035), l’intégrité de la capsule de Bowman (p = 0,014), la présence de croissants (p = 0,019), la fibrose interstitielle (p = 0,03) et l’atrophie tubulaire (p = 0,006). Sur le plan thérapeutique, le traitement par BSRAA semble avoir un effet protecteur (p = 0,01) alors que le recours aux échanges plasmatiques était plutôt un facteur de mauvais pronostic (p = 0,03) (Tableau 4).

**Tableau 4:** Comparaison entre les groupes de patients IRCT et non IRCT

Paramètre	Groupe IRCT	Groupe non IRCT	P
Genre M/F	8/1	10/6	0,13
Fièvre	4	4	0,42
Arthralgies	9	14	0,39
Vomissements	**4**	**1**	**0,022**
Hémorragie digestive	3	1	0,08
Céphalées	2	3	0,88
Convulsion	1	1	0,58
Hématurie macro	5	3	0,09
HTA	3	6	0,49
Protéinurie	8	13	0,45
SN	2	5	0,52
SNA	2	1	0,16
Créatininémie >120 (µmol/l)	**8**	**2**	**0,0004**
Classe PBR			
Classe I (n=5)	**0**	**5**	**0,0001**
Classe II (n=11)	**3**	**8**	
Classe III (n=2)	**0**	**2**	
Classe IV (n=5)	**4**	**1**	
Classe V (n=2)	**2**	**0**	
Prolifération endocap	**6**	**3**	**0,03**
Inflam interst	4	2	0,14
Intégrité caps Bowman	**7**	**13**	**0,01**
Croissants	**7**	**3**	**0,01**
Fibrose interst	**7**	**6**	**0,03**
Atrophie tubulaire	**3**	**1**	**0,006**
Nécrose glom >10%	2	3	0,87
Sclérose glom >10%	**5**	**0**	**0,0002**
Fibrose interst > 10%	**6**	**2**	**0,0049**
Cylindres hématiques	3	4	0,59
Echanges plasmatiques	**2**	**0**	**0,034**
TTT par BSRA	**0**	**8**	**0,011**

IRCT : insuffisance rénale chronique terminale, M : masculin, F : féminin, HTA : hypertension artérielle, SN : syndrome néphrotique, SNA : syndrome néphrétique aigu, endocap : endocapillaire, inflam : inflammation, interst : interstitielle, glom : glomérulaire, TTT : traitement, BSRA : bloqueur du système rénine angiotensine, Macro : macroscopique, PBR : ponction biopsie rénale

## Discussion

Cette étude décrit les aspects cliniques et évolutifs de la néphropathie de la vascularite à IgA. Les principaux facteurs pronostiques identifiés étaient l’atteinte digestive, l’insuffisance rénale initiale et la sévérité des lésions histologiques rénales. Toutefois, il convient de souligner que nous avons étudié un groupe de patients sélectionnés dont l’atteinte rénale liée à la V IgA était suffisamment grave pour justifier la pratique d’une biopsie rénale. Chez l’enfant comme chez l’adulte, la maladie est plus fréquente chez les patients de sexe masculin [1, 12, 13]. Nos données étaient comparables à celles de la littérature avec une nette prédominance masculine. Il n’existe aucun signe biologique spécifique de la maladie [1]. Le taux sérique d’IgA est élevé dans 60% des cas mais cela ne constitue en aucun cas un argument formel pour affirmer le diagnostic [3, 14, 15]. Cette analyse a été peu réalisée dans notre étude avec un taux élevé dans seulement deux cas.

Le pronostic de la maladie à court terme dépend de la sévérité de l’atteinte digestive, mais à long terme elle est tributaire de l’atteinte rénale [3]. L’incidence de l’IRCT était de 36% dans notre population avec une survie rénale à la fin du suivi de 57% témoignant du mauvais pronostic rénal de nos malades contrairement aux autres études publiées où le pronostic était meilleur avec une incidence de l’IRCT beaucoup plus faible [3, 7]. Nous avons retrouvé que les lésions histologiques étaient plus prédictives du pronostic rénal que la présentation clinique comprenant les lésions purpuriques et l’atteinte articulaire. La présence d’une hématurie macroscopique et la protéinurie n’étaient pas aussi des facteurs prédictifs du pronostic rénal dans notre population ainsi que la présence d’un syndrome néphrotique contrairement aux données de la littérature [14, 16-18]. Certains auteurs ont identifié l’HTA au diagnostic comme un facteur pronostique ce qui n’a pas été objectivé dans notre série [19, 20]. On a retrouvé par contre que l’atteinte digestive se traduisant par les vomissements était un facteur pronostique. La présence d’une insuffisance rénale initiale était aussi un important facteur pronostique dans notre étude ce qui est comparable aux données de la littérature [3, 7, 20-22]. Les principaux autres facteurs pronostiques identifiés dans notre série étaient les lésions histologiques et notamment la classification glomérulaire. L’atteinte histologique la plus fréquemment retrouvée dans notre population était la classe II au contraire d’autres études où c’est plutôt la classe III qui est la plus fréquente [3].

La grande disparité des lésions histologiques dans la littérature est probablement due aux indications différentes de la biopsie rénale d’une équipe à une autre [1, 3, 23]. Nous avons indiqué une biopsie rénale chez nos patients devant des anomalies urinaires à type de protéinurie ou hématurie et devant l’existence d’une insuffisance rénale. On a démontré que les lésions histologiques chroniques telles que le pourcentage de fibrose interstitielle ou de sclérose glomérulaire étaient des facteurs de mauvais pronostic. Ces lésions sont souvent décrites au cours d’autres néphropathies glomérulaires et particulièrement la néphropathie à IgA [1, 3].

Le traitement de la V IgA est le plus souvent symptomatique. Ainsi, nous avons trouvé que le traitement par BSRA était un facteur pronostique dans notre population ce qui conforte l’effet néphroprotecteur de cette classe thérapeutique. L’utilisation de traitements plus spécifiques (stéroïdes et/ou immunosuppresseurs), chez les patients ayant une forme clinique préoccupante, reste encore controversée [1, 3, 24, 25]. Notre analyse univariée n’a pas montré de bénéfice d’un traitement spécifique sur la fonction rénale. Le recours aux échanges plasmatiques était un facteur de mauvais pronostic dans notre étude. Toutefois, des études récentes ont retrouvé un effet bénéfique de ce traitement seul ou en association avec la corticothérapie dans les formes sévères de V IgA [26, 27]. L’importance des lésions histologiques irréversibles telles que la fibrose interstitielle et la sclérose glomérulaire étant des indicateurs de mauvais pronostic, rendent l’efficacité thérapeutique douteuse. La transplantation est envisageable au cours de cette affection. Toutefois, un délai court entre la transplantation rénale et les premiers signes cliniques de la maladie est un facteur de risque à prendre en compte [1, 3]. Ainsi, parmi les deux patients ayant bénéficié d’une greffe rénale dans notre étude, une récidive de la maladie a été observée chez le patient ayant été greffé seulement six mois après le passage en IRCT avec une évolution ultérieure fatale. Notre étude présente toutefois certaines limites et notamment l’effectif relativement faible ne nous permettant pas de réaliser une étude multivariée.

## Conclusion

L’insuffisance rénale initiale et la sévérité des lésions histologiques étaient les principaux facteurs pronostiques identifiés dans notre étude. Cette complication doit être recherchée systématiquement afin de prévenir ou de ralentir l'évolution vers l'insuffisance rénale terminale. Le traitement est essentiellement symptomatique et devant le caractère rétrospectif de cette étude et son effectif assez faible nous ne pouvons recommander le non recours au traitement spécifique dans l’attente d’études randomisées comparatives.

## Conflits d’intérêts

Les auteurs ne déclarent aucun conflit d’intérêts.
